# Blood biomarkers for neuroaxonal injury and astrocytic activation in chemotherapy-induced peripheral neuropathy

**DOI:** 10.2340/1651-226X.2024.39895

**Published:** 2024-08-05

**Authors:** Jamila Adra, Daniel Giglio, Per Karlsson, Henrik Zetterberg, Zakaria Einbeigi

**Affiliations:** a Department of Oncology, Institute of Clinical Sciences, Sahlgrenska Academy, University of Gothenburg, Sweden; b Department of Oncology, Sahlgrenska University Hospital, Gothenburg, Sweden; c Department of Psychiatry and Neurochemistry, Institute of Neuroscience and Physiology, the Sahlgrenska Academy at the University of Gothenburg, Mölndal, Sweden; d Clinical Neurochemistry Laboratory, Sahlgrenska University Hospital, Mölndal, Sweden; e Department of Neurodegenerative Disease, UCL Institute of Neurology, Queen Square, London, UK; f UK Dementia Research Institute at UCL, London, UK; g Hong Kong Center for Neurodegenerative Diseases, Clear Water Bay, Hong Kong, China; h Wisconsin Alzheimer’s Disease Research Center, University of Wisconsin School of Medicine and Public Health, University of Wisconsin-Madison, Madison, WI, USA; i Department of Medicine and Oncology, Södra Älvsborgs Hospital, Borås, Sweden

**Keywords:** Breast cancer, chemotherapy, neurofilament, neuropathy

## Abstract

**Background and purpose:**

Chemotherapy-induced peripheral neuropathy (CIPN) is a troublesome side effect in patients exposed to taxanes in the treatment of cancer and may affect quality of life dramatically. Here we assessed whether serum levels of neurofilament light (NfL) and tau (two neuroaxonal injury biomarkers) and glial fibrillary acidic protein (GFAP, a biomarker for astrocytic activation) correlate with the development of CIPN in the adjuvant setting of early breast cancer.

**Materials and methods:**

Using ultrasensitive single molecule array technology, serum levels of NfL, GFAP, and tau were measured before and every 3 weeks in 10 women receiving adjuvant EC (epirubicin 90 mg/m² and cyclophosphamide 600 mg/m²) every 3 weeks × 3, followed by weekly paclitaxel 80 mg/m² × 9–12 weeks after surgery due to early breast cancer. CIPN was graded according to the NCI Common Terminology Criteria for Adverse Events (CTCAE v5.0) and the questionnaire EORTC QLQ CIPN-20.

**Results:**

Serum levels of GFAP increased successively during cycles of EC. NfL increased instead in response to the treatment of paclitaxel. NfL and GFAP continued to rise throughout exposure of cumulatively higher doses of paclitaxel and were reduced 3 months after the end of chemotherapy. Serums levels of tau were marginally affected by exposure to chemotherapy. Women with worse symptoms of CIPN had higher concentrations of NfL than women with mild symptoms of CIPN.

**Interpretation:**

NfL and GFAP are promising biomarkers to identify women at risk of developing CIPN. Larger prospective studies are now needed.

## Introduction

Taxanes, including paclitaxel and docetaxel, are given in the adjuvant setting of breast cancer treatment as well as in the metastatic setting. In the adjuvant setting when most patients have a long-life expectancy, it is important that treatment does not cause difficult and irreversible side effects. Chemotherapy-induced peripheral neurotoxicity (CIPN) is one of the most frequently occurring side effects associated with taxane use in cancer treatment. It may lead to a decline in daily activities and quality of life (QoL). This can sometimes be chronic [1–3]. CIPN may also lead to dose reductions, longer time intervals between treatments and sometimes the need of ending chemotherapy sooner than planned [4]. The reported frequency of CIPN associated with taxane use varies between studies. Studies in patients with metastatic breast cancer show that CIPN induced by taxanes occurred in 4%–30% of cases, where the dose and dose interval of paclitaxel correlated with the severity of CIPN [5].

Serum levels of neurofilaments (Nfs) have recently been shown to constitute biomarkers of axonal injury due to their exclusive expression in axons, where they play a critical role in structural stability [6]. Nf subunits are biologically classified according to their molecular weight as Nf light (NfL, 68kDa), Nf medium (NfM, 160 kDa), and Nf heavy (NfH, 200 kDa) [7]. Nfs are released into extracellular fluid upon axonal injury and can be detected in cerebrospinal fluid and blood [8]. Over the last years, several studies have shown that NfL may constitute a biomarker for several different neurological diseases such as Alzheimer’s disease [9], multiple sclerosis [10], amyotrophic lateral sclerosis, and Parkinson’s disease [11], as well as peripheral neuropathies, including Charcot-Marie-Tooth disease [12, 13], neuropathy in hereditary transthyretin amyloidosis [14], and chronic inflammatory demyelinating polyradiculoneuropathy [15, 16]. Glial fibrillary acidic protein (GFAP) is a monomeric intermediate filament protein found in the astroglial cytoskeleton and is found mainly in the central nervous system (CNS). GFAP is released after cell death or injury and/or astrocytic activation, such as after traumatic brain injury [17]. Tau belongs to the microtubule-associated proteins family and is very important for the stabilization of microtubules, as well as in the pathogenesis of various neurodegenerative diseases [18].

Previous studies have shown that NfL could be a useful biomarker of axonal degeneration in a rat model of vincristine-induced peripheral neurotoxicity [19]. Rats that are treated with paclitaxel also show an increase of NfL serum levels that correlate with the severity of axonal damage [20]. Several studies have shown that the plasma concentration of NfL increases in women treated with paclitaxel against breast and gynecological cancer [21–23]. Few studies have been conducted assessing the time-dependent dynamics of plasma levels of NfL, GFAP, and tau throughout the course of chemotherapy against cancer.

The aim of our study was to assess the dynamics of serum levels of NfL, GFAP, and tau in women exposed to adjuvant chemotherapy in early breast cancer and whether serum levels of NfL, GFAP, and tau predict and/or correlate with the severity of CIPN.

## Methods

### Recruitment, cohort, and therapy

Ten patients who had gone through surgery due to early breast cancer and who were recommended adjuvant chemotherapy were recruited to the study at the Department of Oncology, Sahlgrenska University Hospital, Gothenburg, Sweden in 2020. The patients were asked consecutively to participate in the study without selection. Most patients accepted participation with the exception of a few. The adjuvant chemotherapy was recommended according to current national guidelines, that is, three cycles of epirubicin (75–90 mg/m^2^)-cyclophosphamide (600 mg/m^2^; EC), followed by 9 or 12 weekly doses of paclitaxel (80 mg/m^2^). Seventeen doses of trastuzumab (1^st^ dose 8 mg/kg, from 2^nd^ dose 6 mg/kg) were given every 3 weeks to patients with HER2-positive breast cancer. Patients could have undergone either breast-conserving surgery (BCS) or modified radical mastectomy (MRM). Both node negative (N0) and node positive (N+) were allowed to be included. Exclusion criteria were previously given chemotherapy or other diseases with neurological impairment (e.g. diabetes). Adjuvant radiotherapy was given according to guidelines as well as adjuvant endocrine therapy. The patients were followed by regular doctor’s appointments by the same standard schedule as for all patients at our clinic.

### Measurement of biomarkers

**Figure 1:**
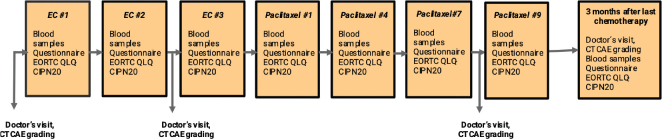
Flow chart.

Figure 1 illustrates a flowchart of collecting blood samples of peripheral venous blood. Blood samples were collected at baseline before the start of chemotherapy, as well as every 3 weeks throughout the entire period of chemotherapy. A final blood sample was taken 3 months after the ended chemotherapy. Samples were collected in EDTA tubes that were centrifuged at 2200 × g for 10 min at room temperature. Following centrifugation, the plasma supernatant was separated and aliquoted in 0.5 mL portions in cryotubes that were stored in −80°C prior to analysis. NfL, GFAP, and tau concentrations were measured using the Neurology 4-plex B kit on a single molecule array (Simoa) HD-X Analyzer according to the instructions from the manufacturer (Quanterix, Billerica, MA). All measurements were performed in one round of experiments using one batch of reagents by board-certified laboratory technicians who were blinded to clinical data. Longitudinal samples from the same individual were measured side by side on the same plate to minimize variation. Intra-assay coefficients of variation were 8.8%–14% for NfL, 7.6%–15% for GFAP, and 2.4%–6.9% for tau.

### Assessment of CIPN

Two methods were used to grade peripheral neuropathy, that is, with questionnaire (EORTC QLQ-CIPN20) and physician assessment (CTCAE v5.0). Every 3 weeks during chemotherapy treatment (at the same time as blood samples were collected) and 3 months after chemotherapy treatment had ended, patients filled out the self-esteemed form EORTC QLQ-CIPN20. The form is obtained from the European Organisation for Research and Treatment of Cancer, EORTC. The QLQ-CIPN20 contains 20 items assessing sensory (9 items), motor (8 items), and autonomic symptoms (3 items). Erectile dysfunction is excluded as it is not applicable to women. Using a 4-point Likert scale (1 = ‘not at all’, 2 = ‘a little’, 3 = ‘quite a bit’, and 4 = ‘very much’), individuals indicate the degree to which they have experienced sensory, motor, and autonomic symptoms during the past week. The scores were linearly converted to a 0–100 scale, with higher scores indicating more symptoms [24].

The patients were clinically assessed by a medical oncologist according to clinical practice. This took place at the start of chemotherapy, after three cycles of EC (at 9 weeks from baseline), after 7 weekly doses of paclitaxel (at 15 weeks from baseline) and 3 months after chemotherapy had ended. The grade of peripheral neuropathy was assessed using the National Cancer Institute Common Terminology Criteria for Adverse Events (NCI CTCAE v5.0) and was graded 0–4.

### Statistics

Values of biomarkers are given as percentages of baseline values. The extra-sum-of-squares F test was used to compare curves between low-grade and high-grade neuropathy. A p-value of less than 0.05 was considered significant.

## Results

Table 1 shows the baseline characteristics of the studied cohort. The majority had estrogen receptor-positive (ER+) disease. All patients had undergone axillary surgery, either sentinel node-biopsy (*n* = 7) or axillary lymph node dissection (*n* = 3). Three patients ended paclitaxel treatment earlier than initially planned. One patient received eight of nine planned doses due to severe neuropathy. One patient received six doses and one patient received eight of nine planned doses due to leukopenia. Four patients had some level of dose reduction of paclitaxel during the chemotherapy treatment. Two patients had dose reduction because of increasing neuropathy, one patient due to leukopenia and one patient due to hepatic impairment. The latter patient also received only one dose of EC due to impaired liver function.

**Table 1:** Patient characteristics at baseline.

Characteristics	No. (%)
**Age**	
Median	61
Range	49–73
**Female sex no (%)**	10 (100)
**Type of breast surgery *n* (%)**	
Mastectomy	5 (50)
Lumpectomy	5 (50)
**Type of axilla surgery *n* (%)**	
Sentinel-node biopsy	7 (70)
Axillary lymph node dissection	3 (30)
**Metastasis status at baseline**	
No metastases	4 (40)
Lymph node metastases	6 (60)
Distant metastases	0 (0)
**Biologic subtype**	
ER+, HER2 negative	7 (70)
HER2 positive	2 (20)
Triple negative	1 (10)

### Biomarkers

**Figure 2:**
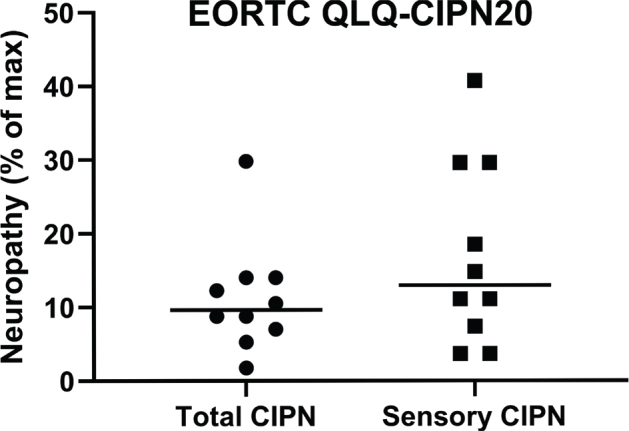
Shows the highest score for each patient using the EORTC QLQ-CIPN20 questionnaire, all items and sensory items separately. Cut-off was set to get five patients in each group, low-grade vs high-grade CIPN. When observing sensory items separately, more patients had a higher score.

Plasma NfL, GFAP, and tau concentrations were measured in all patients at baseline. Large interindividual variations in baseline concentration could be observed (Supplementary Table S1, from 4.44 to 65.2 pg/mL). NfL slightly increased during the initial three EC cycles. In one patient, biomarkers were not measured before EC cycles 2 and 3 since EC cycles 2 and 3 were not given due to hepatic impairment. At the first administered dose of paclitaxel, a prominent increase of NfL occurred in all 10 patients (Supplementary Table S1, Figure 2A). After dose 8 of paclitaxel, an average increase of 2,630% of baseline was noted. Three months after ending chemotherapy, the average concentration of NfL decreased to 471% of baseline. At baseline, the average concentration of GFAP was 81.6 pg/mL. An average increase of GFAP could be seen during chemotherapy treatment although not in every patient (Supplementary Table S2). After dose 8 of paclitaxel, the average concentration of GFAP was 145% of baseline. Three months after chemotherapy had ended, the average concentration of GFAP was 109% of baseline. At baseline, the average concentration of tau was 2.10 pg/mL. An increase in tau tended to occur during paclitaxel treatment (Supplementary Table S3).

### Patient report

All 10 patients reported CIPN at some point during chemotherapy, that is, score 2 or higher on the EORTC QLQ-CIPN20 questionnaire. The most reported symptom was tingle/painful itching in fingers and/or toes. The scores were linearly converted to a 0–100 scale in order to obtain a percentual score. The total percentual score of all 19 questions for each patient throughout chemotherapy is shown in Supplementary Table S4. When looking specifically at the 9 questions that ask for sensory symptoms, a higher percentual score was noted in some patients compared to the total percentual score (Supplementary Table S5). We divided the patients into two groups, above or below the median value of the EORTC QLQ-CIPN20 score (Figure 2). For total CIPN, the median value was 8.95. For sensory CIPN, the median value was 12.6 on a 0–100 scale.

**Figure 3:**
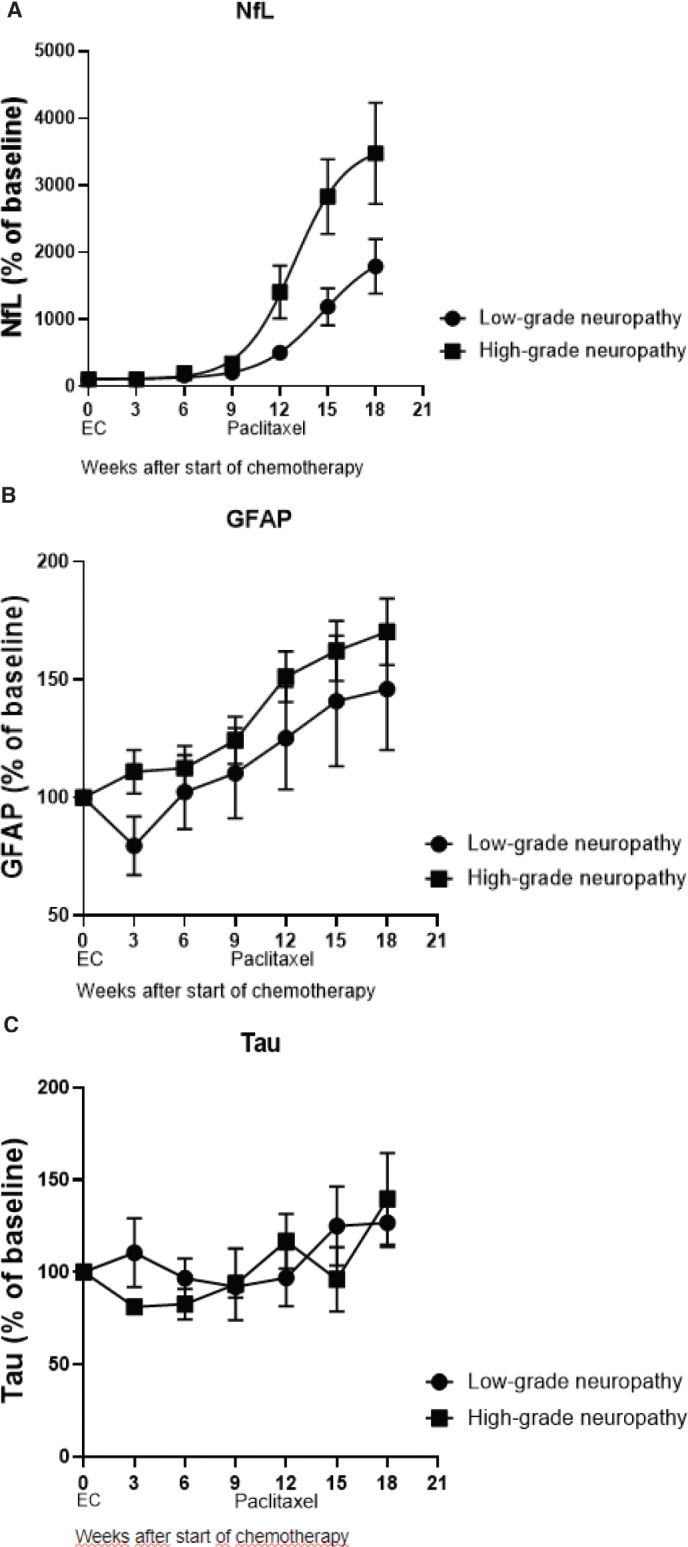
(A–C) Demonstrate the average percentual change in plasma concentration of NfL, GFAP, and tau during time of chemotherapy treatment, showing patients with low-grade and high-grade CIPN separately.

**Figure 4:**
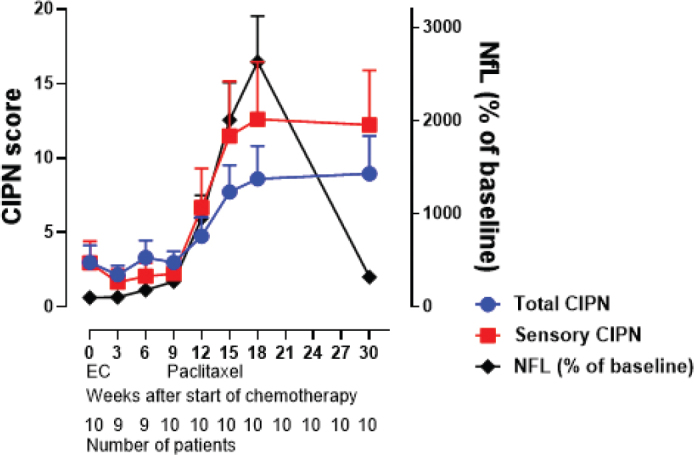
Demonstrates average percentual score on EORTC QLQ CIPN20 for all patients throughout treatment chemotherapy, regarding all items (global average score) as well as sensory items (sensory average score), compared to NfL plasma concentration. Number of patients is marked under each time plot.

In the high-grade CIPN group, NfL was significantly higher than the low-grade CIPN group (*p* < 0.0001; Figure 3A). GFAP increased in response to cumulative doses of paclitaxel; however, no differences were observed between the low-grade and high-grade CIPN groups (*p* = 0.25; Figure 3B). Plasma levels of tau were not significantly affected by the administration of chemotherapy (Figure 3C). The average percentual score from EORTC QLQ CIPN20 increased throughout treatment with paclitaxel. The comparison to the increase of NfL plasma concentration is shown in Figure 4. Correlations between all biomarkers and EORTC QLQ scores, both total and sensory, are shown in Supplementary Table S6.

### Physician’s assessment

Eight patients were assessed as CTCAE-score 0, that is, no symptoms, at baseline. For the remaining two patients, no assessment was documented at baseline. After three doses of EC, all 10 patients were assessed as grade 0. After seven doses of weekly paclitaxel, four patients were still assessed as grade 0, five as grade 1, that is, loss of deep tendon reflexes or paresthesia, and one patient as grade 2, that is, moderate symptoms, limiting instrumental activity of daily life (ADL). Unfortunately, only three patients had documented assessment 3 months after chemotherapy had ended. These three patients were all scored as grade 1. In comparison with the last previous grading, one patient went up from grade 0, one patient remained as grade 1, and the third patient downgraded from grade 2.

## Discussion

The present study demonstrated that serum concentrations of NfL and GFAP increased in response to administration of paclitaxel. We also showed that plasma level of NfL was correlated with the severity of CIPN and clearly decreased in all patients 3 months after the last cycle of paclitaxel. In particular, sensory symptoms of CIPN were affected in those patients who had a more rapid increase and later a higher concentration of NfL. However, even though an increase in GFAP concentration could be observed during the administration of paclitaxel, no clear correlation was seen with the severity of CIPN. As GFAP is not present outside the CNS, this could give an indication that the plasma level of GFAP does not affect peripheral symptoms in the same extent that Nfs do. Tau seems to have little implication in the severity of CIPN, even though an increase tended to occur during the treatment of paclitaxel.

CIPN is a side effect with a severe impact on QoL [25]. At the same time, it is difficult to measure in an objective and standardized way and, at present, there is no good way to predict if the symptoms will be long-lasting for patients. Our findings are in line with a recently published article showing that serum levels of NfL in patients under treatment with carboplatin-paclitaxel correlate with the severity of CIPN [26]. Measuring NfL may add another tool in the assessment of CIPN. We show that these biomarkers already increase in plasma after the first dose of paclitaxel, and we observed a time-dependent increase in plasma levels of NfL and GFAP throughout treatment cycles. Hence, these biomarkers seem to constitute sensitive biomarkers to detect peripheral neurotoxicity and correlate with the clinical observation that cumulative doses of paclitaxel lead to higher neurotoxicity. These biomarkers may also be important to help predicting which patients who are at risk of developing chronic CIPN [26]. These biomarkers may be useful in both the adjuvant setting and in the palliative setting where a quick increase in plasma levels of the biomarkers could indicate worse CIPN and, hence, give the physician a possibility to modify the treatment before CIPN gets too severe and painful for the patient.

We could see that six patients graded CIPN grade 3–4 on the EORTC QLQ CIPN20 questionnaire (which means ‘quite a bit’ and ‘very much’). Only one patient was graded 2 (moderate symptoms) on the CTCAE by the physician, while the rest of the patients were graded 0–1. The grades are not fully comparable, and some data were missing from the CTCAE score by the physician. This makes it difficult to come to any clear conclusion whether CIPN was generally underestimated by the physician in our study. The fact that physicians underreport and underestimate the severity of CIPN symptoms compared with patients has been shown in one other study [27], supporting the importance of patient-reported measured outcomes.

Strengths of our study were that we examined the time-dependent dynamics of biomarkers throughout the course of adjuvant chemotherapy, as well when treatment had ended. The data show that plasma concentration of NfL starts to increase immediately when paclitaxel treatment commences and decreases when paclitaxel ends. We also used several different methods to measure CIPN in participating patients. This reduces the risk of bias. A weakness of our study was its small size with only 10 patients included. We could observe that the group who reported worse symptoms correlated to CIPN had a higher NfL plasma level concentration, but there are too few patients to determine whether the differences across symptom severity groups were significant. Future larger studies to assess the validity of NfL to identify women with CIPN are needed. We also had limited follow-up time to 30 weeks after start of chenotherapy. Given that the apparent elimination half-life of NfL is around 40 days [28]*,* the observation that NfL remains slightly elevated at week 30 suggests that there may be lingering axonal injury that has not resolved completely in these patients during the observation period. Studies with even longer follow-up are needed to examine the clinical relevance of this finding more closely.

## Supplementary Material

Blood biomarkers for neuroaxonal injury and astrocytic activation in chemotherapy-induced peripheral neuropathy

## Data Availability

The data that support the findings of this study are available on request from the corresponding author, JA.
