# Supplementary material for: Blood biomarkers for neuroaxonal injury and astrocytic activation in chemotherapy-induced peripheral neuropathy
Source: Acta Oncol. 2024 Aug 5;63:39895. doi: 10.2340/1651-226X.2024.39895 (PMC11332453; doi:10.2340/1651-226X.2024.39895)
Supplement: Blood biomarkers for neuroaxonal injury and astrocytic activation in chemotherapy-induced peripheral neuropathy [file AO-63-39895-s1.pdf]

Supplementary material has been published as submitted. It has not been copyedited, or typeset by Acta Oncologica

Table S1 NfL plasma concentration during chemotherapy

	NfL pg/mL							
	baseline/ *EC1	EC2	EC3	**Pacli1	Pacli4	Pacli7	Pacli9	3 months after chemotherapy
Pat 1	14.6	13.2	20.8	34.7	90.9	221	388	45.7
Pat 2	7.77	9.25	15.6	25.2	53.8	103	175	28.5
Pat 3	8.86	***	***	5.42	15.3	45.6	80.3	15.1
Pat 4	13.8	11.7	25.8	31.4	125	325	772	24.6
Pat 5	10.5	12.9	24	44.2	147	347	484	55.5
Pat 6	8.24	7.49	12.2	16.8	34	49.6	58.4	19
Pat 7	13	12.2	23.4	40.6	342	496	312	39.3
Pat 8	4.44	5.67	12.4	28.5	79.2	169	151	23.3
Pat 9	65.2	52.9	67.5	68.6	201	568	887	227
Pat 10	7.01	8.09	10.6	13.5	42.1	138	170	17.6
Average	15.3	14.8	23.6	30.9	113	246	348	49.6

Footnote: *EC=epirubicin/cyclophosphamide ** Pacli=paclitaxel
***Pat 3 did not receive EC cycle 2 and 3 due to hepatic impairment.

Table S2 GFAP plasma concentration during chemotherapy

	GFAP pg/mL							
	baseline/ *EC1	EC2	EC3	**Pacli1	Pacli4	Pacli7	Pacli9	3 months after chemotherapy
Pat 1	168	164	193	210	161	260	286	191
Pat 2	156	119	113	109	152	137	137	113
Pat 3	105	***	***	40.9	109	76.3	91	80.5
Pat 4	78.8	64.1	76.2	82.4	98.9	96.3	159	95.7
Pat 5	58.4	71.7	66.5	89.6	102	84.4	116	63.3
Pat 6	42.4	31	34.6	38.2	50.1	69.8	70.1	43.4
Pat 7	68.2	89.2	80.1	73.4	118	130	92.9	77.1
Pat 8	41	50.2	59.1	58.8	52.1	75.3	72.2	63.8
Pat 9	70.3	68.8	63.8	79.2	110	120	97.8	100
Pat 10	27.5	30.9	38.7	43.1	58	61.9	60.6	59.3
Average	81.6	76.5	80.6	82.4	101	111	118	88.7

Footnote: *EC=epirubicin/cyclophosphamide **Pacli=paclitaxel
***Pat 3 did not receive EC cycle 2 and 3 due to hepatic impairment.

Table S3 Tau plasma concentration during chemotherapy

	Tau pg/mL							
	Baseline/ *EC1	EC2	EC3	**Pacli1	Pacli4	Pacli7	Pacli9	3 months after chemotherapy
Pat 1	1.58	1.15	1.54	1.43	1.28	1.82	2.64	0.77
Pat 2	1.16	1.62	0.8	1.02	1.62	2.21	1.61	1.71
Pat 3	2.54	***	***	2.19	1.24	1.97	2.61	3.19
Pat 4	1.62	1.1	1.72	1.76	2.33	2.04	3.42	2.94
Pat 5	1.69	1.3	1.34	1.13	1.95	1.03	2.78	2.6
Pat 6	2.2	1.85	2.18	1.77	2.19	1.87	2.06	2.23
Pat 7	4.04	3.32	3.36	2.82	4.15	1.92	3.22	4.94
Pat 8	1.47	1.28	0.81	2.39	2.23	1.94	2.28	1.34
Pat 9	2.07	1.89	1.84	1.22	1.44	2.35	1.81	2.8
Pat 10	2.58	3.74	3.12	2.97	2.97	4.03	3.38	3.46
Average	2.10	1.92	1.86	1.87	2.14	2.12	2.58	2.60

Footnote: *EC=epirubicin/cyclophosphamide **Pacli=paclitaxel
***Pat 3 did not receive EC cycle 2 and 3 due to hepatic impairment.

Table S4 Patients score EORTC QLQ CIPN20, %, all items

	Baseline/ *EC1	EC2	EC3	**Pacli1	Pacli4	Pacli7	Pacli9	3 months after chemotherapy
Pat 1	5.26	3.51	7.02	5.26	5.26	8.77	5.26	7.02
Pat 2	0	1.75	5.26	1.75	5.26	5.26	5.26	7.02
Pat 3	3.51	***	***	3.51	3.51	5.26	5.26	3.51
Pat 4	1.75	0	0	0	0	0	8.77	5.26
Pat 5	0	5.26	8.77	7.02	0	14	26.3	29.8
Pat 6	1.75	0	0	1.75	1.75	0	0	0
Pat 7	1.75	0	5.26	1.75	10.5	14	10.5	10.5
Pat 8	1.75	3.51	0	5.26	12.3	14	10.5	8.77
Pat 9	1.75	3.51	3.51	3.51	3.51	3.51	5.26	10.5
Pat 10	12.3	1.75	0	0	5.26	12.3	8.77	7.02

Footnote: *EC=epirubicin/cyclophosphamide **Pacli=paclitaxel
***Pat 3 did not receive EC cycle 2 and 3 due to hepatic impairment.

Table S5 Patients score EORTC QLQ CIPN20, %, sensory scale items

	Baseline/ *EC1	EC2	EC3	**Pacli1	Pacli4	Pacli7	Pacli9	3 months after chemotherapy
Pat 1	3.7	0	0	0	3.7	11.1	3.7	11.1
Pat 2	0	0	0	0	3.7	3.7	7.41	7.41
Pat 3	0	*	*	0	0	3.7	3.7	0
Pat 4	3.7	0	0	0	0	0	14.8	11.1
Pat 5	0	3.7	3.7	3.7	0	22.2	40.7	37
Pat 6	0	0	0	3.7	3.7	0	0	0
Pat 7	0	0	7.41	0	14.8	29.6	18.5	18.5
Pat 8	3.7	7.41	0	7.41	25.9	29.6	22.2	18.5
Pat 9	3.7	3.7	3.7	3.7	3.7	3.7	3.7	18.5
Pat 10	14.8	0	0	0	11.1	11.1	11.1	0

Footnote: *EC=epirubicin/cyclophosphamide **Pacli=paclitaxel
***Pat 3 did not receive EC cycle 2 and 3 due to hepatic impairment.

Table S6 Correlations between biomarkers and EORTC QLQ CIPN20 score, both total and sensory. Pearson correlations coefficients

	CIPNtot	CIPNs~s	NfL	GFAP	Tau
CIPNtot*	1.0000				
CIPNsens**	0.8721	1.0000			
NfL	0.2714	0.3912	1.0000		
GFAP	0.1013	0.0202	0.3265	1.0000	
Tau	0.1633	0.2142	0.1570	-0.2058	1.0000

Footnote: *tot=total, **sens=sensory

EORTC QLQ-CIPN20 questionnaire

1. Did you have tingling fingers or hands?[a]
2. Did you have tingling toes or feet?[a]
3. Did you have numbness in your fingers or hands?[a]
4. Did you have numbness in your toes or feet?[a]
5. Did you have shooting or burning pain in your fingers or hands?[a]
6. Did you have shooting or burning pain in your toes or feet?[a]
7. Did you have cramps in your hands?[b]
8. Did you have cramps in your feet?[b]
9. Did you have problems standing or walking because of difficulty feeling the ground under your feet?[a]
10. Did you have difficulty distinguishing between hot and cold water?[a]
11. Did you have a problem holding a pen, which made writing difficult?[b]
12. Did you have difficulty manipulating small objects with your fingers (for example, fastening small buttons)?[b]
13. Did you have difficulty opening a jar or bottle because of weakness in your hands?[b]
14. Did you have difficulty walking because your feet dropped downwards?[b]
15. Did you have difficulty climbing stairs or getting up out of a chair because of weakness in your legs?[b]
16. Were you dizzy when standing up from a sitting or lying position?[c]
17. Did you have blurred vision?[c]
18. Did you have difficulty hearing?[a]

Please answer the following question only if you drive a car

19. Did you have difficulty using the pedals?[b]

Please answer the following question only if you are a man

20. Did you have difficulty getting or maintaining an erection?[c]

Items have been renumbered from the original QLQ-CIPN20 instrument

[a]Sensory scale items
[b]Motor scale items
[c]Autonomic scale items
